# Fabricated CAD/CAM Post-Core Using Glass Fiber-Reinforced Resin Shows Innovative Potential in Restoring Pulpless Teeth

**DOI:** 10.3390/ma14206199

**Published:** 2021-10-19

**Authors:** Naoko Suzaki, Satoshi Yamaguchi, Eriko Nambu, Ryousuke Tanaka, Satoshi Imazato, Mikako Hayashi

**Affiliations:** 1 Department of Restorative Dentistry and Endodontology, Osaka University Graduate School of Dentistry, 1-8 Yamadaoka, Suita 565-0871, Osaka, Japan; naoko-suzaki@dent.osaka-u.ac.jp (N.S.); r.tanaka3570@dent.osaka-u.ac.jp (R.T.); 2 Department of Biomaterials Science, Osaka University Graduate School of Dentistry, 1-8 Yamadaoka, Suita 565-0871, Osaka, Japan; yamagu@dent.osaka-u.ac.jp (S.Y.); imazato@dent.osaka-u.ac.jp (S.I.); 3 Division of Medical Information, Osaka University Dental Hospital, 1-8 Yamadaoka, Suita 565-0871, Osaka, Japan; nambu-e@office.osaka-u.ac.jp

**Keywords:** fiber-reinforced resin composites, CAD/CAM, indirect post-core

## Abstract

The prevention of root fractures of pulpless teeth is an important clinical issue to maintain healthy teeth through lifetime. The aim of this study was to examine a clinically effective treatment method for strengthening vulnerable pulpless teeth using CAD/CAM (computer-aided design/computer-aided manufacturing) fiber-reinforced post-core by conducting a fracture resistance test. A post-core made with a fiber-reinforced resin disk TRINIA (TR, SHOFU, Kyoto, Japan) was fabricated using a CAD/CAM system. The fiber-layer orientation of the CAD/CAM post-core was parallel to the axis of the restored tooth. A post-core using a conventional composite and a fiber post (CF) was also prepared. A fracture resistance test of teeth restored with the post-cores and zirconia crowns was conducted using a universal testing machine, and fracture patterns were identified by micro-CT observation. The fracture load of the roots restored with TR was 1555.9 ± 231.8 N, whereas that of CF was 1082.1 ± 226.7 N. The fracture load of TR was 43.8% that was significantly higher than that of CF (Student’s *t*-test, *p* < 0.05). The restored teeth with CAD/CAM resin post-core were found to be repairable even after fracture. These results suggest that the CAD/CAM indirect fiber post-core has the potential to strengthen the vulnerable pulpless teeth.

## 1. Introduction

Root fractures of pulpless teeth are one of the most common causes of teeth loss in patients, who receive regular preventative care for caries and periodontitis [1]. Thus, the prevention of root fractures of pulpless teeth is an important clinical issue to maintain healthy teeth through lifetime. 

In daily clinical practice, we often encounter pulpless teeth with extremely limited remaining tooth structure, owing to the repeated restorative treatments undergone. Reinforcement of the remaining tooth with reliable adhesive materials can be an effective measure to preserve such teeth by preventing root fracture. Indirect post-cores with fiber posts and resin cores are recommended for restoring vulnerable pulpless teeth [2,3,4], since using materials with a modulus of elasticity like dentin is important for preventing root fractures [5].

In the present study, we focused on the fiberglass-reinforced resin disk TRINIA (SHOFU), consisting of 55 wt.% fiberglass woven into a mesh and layered structure, with an epoxy resin matrix. TRINIA is mainly used as a framework for bridges and implant superstructures using CAD/CAM technology [6]. We used TRINIA as CAD/CAM post-cores with an aim to maximize the effect of reinforcement of pulpless teeth with fiber materials by adhering uniformly distributed fiber materials to root dentin.

In our previous study, TRINIA was shown to have distinct anisotropy and that it can be used as a superior restorative material by ensuring specific directions of its fiber mesh layers [7]. The anisotropy of TRINIA is totally different from that of conventional fiber post-cores along to longitudinal axis and possibly support for thin remain dentin. This is the first report where clinical usefulness of a CAD/CAM resin post-core using the fiberglass-reinforced resin disk, TRINIA was tested. The null hypothesis of this study was that fracture resistance of a pulpless restored with a CAD/CAM resin post-core was equal to the one restored with a conventional indirect resin post-core.

## 2. Materials and Methods

Sixteen extracted bovine incisors for 30 months old with an apical size up to 1.3 mm were prepared and stored at 4 °C in distilled water. Bovine teeth were provided from the manufacturer (SHOFU). A fracture resistance test was conducted on bovine incisors with an aim of evaluating clinical usefulness of a CAD/CAM resin post-core. The materials used are presented in Table 1.

**Figure 1:**
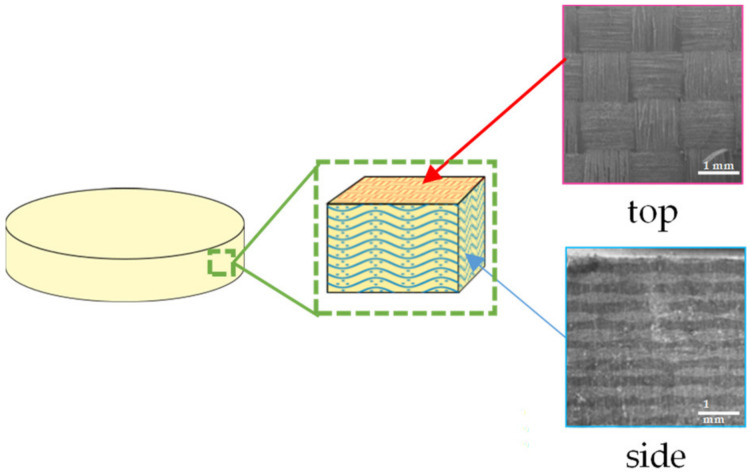
CAD/CAM glass fiber-reinforced resin disk (TRINIA).

A glass fiber-reinforced resin disk (TRINIA) comprised a 45 wt.% epoxy resin matrix and 55 wt.% multi-directionally interlaced glass fibers that aligned the woven layers parallel to the top surface of the disk (Figure 1).

### 2.1. Evaluation of Fracture Resistance of Teeth Restored with CAD/CAM Resin Post-Cores and Zirconia Crowns

#### 2.1.1. Preparation of Teeth

Single-root teeth taken from a 30-month-old bovine were restored with a fiber post-core and a zirconia crown. The teeth were stored in saline at 4 °C and used within 3 months of extraction. The crown of the bovine tooth was removed with a low-speed diamond saw (ISOMET2000, BUEHLER, Lake Bluff, IL, USA) at the cement-enamel junction and the root was adjusted to a length of 15 mm (diameter of major axis: 7.8 ± 0.5 mm, diameter of minor axis: 7.0 ± 0.4 mm) using a rotary grinder (ECOMET III, BUEHLER, Lake Bluff, IL, USA). After the root canal treatments were performed using a K-file (MANI, Tochigi, Japan) up to #60 and irrigation with 2.5% NaOCl (Neo Dental Chemical Products, Tokyo, Japan), the root canal was obturated with gutta-percha points and a canal sealer (Nishika Canal Sealer N, Nippon Dental Chemicals, Yamaguchi, Japan). A post space was prepared to be a depth of 10 mm with 06 taper was prepared using a turbine and diamond points (AR2, GC, Tokyo, Japan) with an air-turbine (Morita, Osaka, Japan). During the preparation, the root thickness of the specimen at the coronal, middle and apical levels was measured at four locations for each 90° circumference using digital calipers (CD-15C, Mitsutoyo, Kanagawa, Japan).

**Figure 2:**
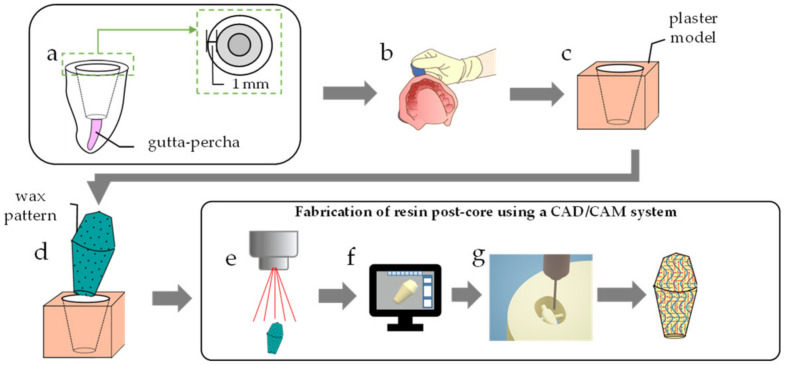
Fabrication of (**a**) CAD/CAM post-core a: Preparing a post hole; (**b**) Taking impression of the prepared tooth; (**c**) Making a working model from the impression; (**d**) Making a wax pattern of a post-core on the working model; (**e**) 3D scanning the working model and the wax pattern; (**f**) Designing a 3D digital post-core; (**g**) Milling TRINIA into a shape of the post-core.

The prepared teeth were then divided into two groups: the TRINIA group and the conventional fiber post group. In the TRINIA group, a post-core made with TRINIA was fabricated using a CAD/CAM system composed of a scanner (S-WAVE scanner D2000, SHOFU) with 5-µm resolution, a software (GO2 dental, SHOFU), and a milling machine (DWX-50, Roland, Shizuoka, Japan) for processing the resin disk (Figure 2). After scanning a wax pattern, and the post-core was produced by the CAD/CAM system by identifying the tooth axis parallel to the TRINIA fiber mesh.

As the control, a conventional fiber post-core was fabricated with a resin core (Beauti Core Flow Paste, SHOFU) and a fiber post (Beauti Core Fiber Post φ = 1.6 mm, SHOFU) on a plaster model.

Each group had eight specimens. The adhesive materials used in this study are shown in Table 1. The surfaces of the post-cores were treated with a primer (HC primer, HC, SHOFU) for 10 s using an applicator brush and dried with gentle air blow for approximately 5 s to evaporate the solvent. The HC primer is characterized by the penetration of the monomer contained into the resin.

A resin cement (ResiCem, SHOFU) was then applied to the root, and the post-core was bonded to the root with light-curing for 10 s using an LED unit (Pencure 2000, Morita, Osaka, Japan).

Next, a full zirconia crown (Aadva Zirconia disk, GC) was bonded to the root restored with a post-core. The post-core was coated with a ResiCem primer and left to dry for 20 s. The zirconia crown, which had been coated with AZ primer (GC) and left to dry for 10 s, was adhered to the core with ResiCem paste fully filled and cured by LED light irradiation for 10 s.

#### 2.1.2. Evaluation of Fracture Resistance

The root surface of the restored tooth was coated with an approximately 200 µm-thick polyvinylsiloxane impression material (Duplicone, SHOFU) to simulate the periodontal ligament. The restored root was embedded in an epoxy resin block (NER814, Nissin EM, Tokyo, Japan) at a depth 2 mm below the cement enamel junction. All specimens were stored at 37 °C with 100% humidity for 24 h before fracture testing.

**Figure 3:**
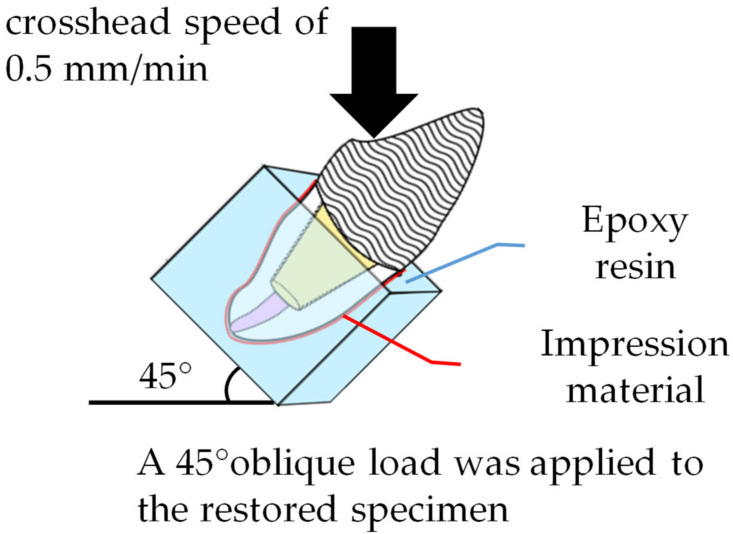
Fracture loads of bovine roots restored with fiber-reinforced post-cores and zirconia crowns.

A 45° oblique load was applied to the center of the palatal surface of the specimen at a crosshead speed of 0.5 mm/min with a universal testing machine (AUTOGRAPH AG-IS 20-KN, Shimadzu, Kyoto, Japan) until fracture (Figure 3).

The results were analyzed by conducting Student’s *t*-test at a significance level of 95% (IBM SPSS Statistics Version 22, IL, USA).

After the fracture test, the specimen was photographed using a micro-CT (R_mCT2, RIGAKU, Tokyo, Japan) to determine the crack propagation areas. Crack propagation was classified into three categories: cervical (fractures extending within 1/3 the length of the root, longitudinally from the cervical portion), middle (fractures extending between 1/3 and 2/3 from the cervical to apical portion), and apical (fracture extending longitudinally to the apical third of the root).

## 3. Results

**Figure 4:**
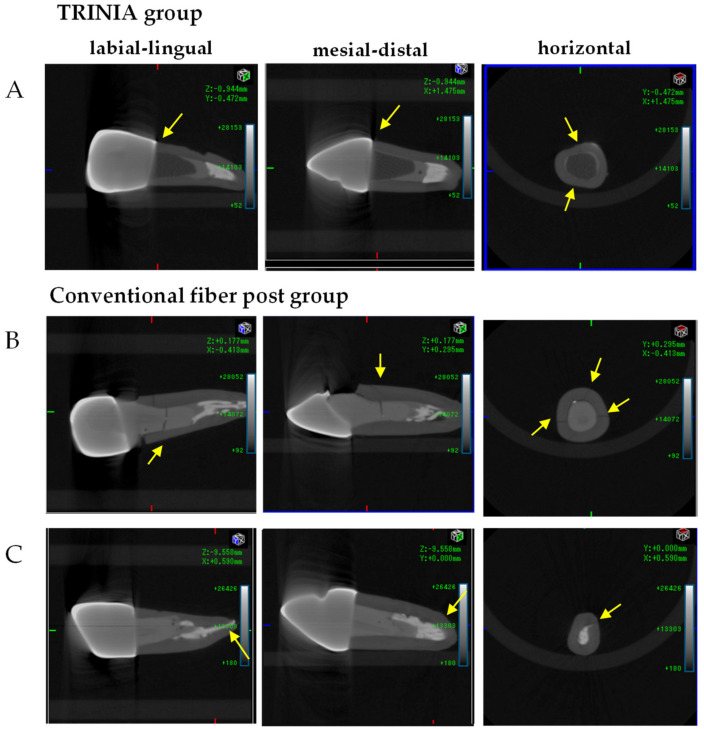
Micro-CT images of the bovine roots restored with fiber-reinforced post-cores and zirconia crowns. In the TRINIA group (**A**), all the specimens were fractured in the cervical and middle areas. In the conventional fiber post group, 37.5% of fractures occurred in the cervical and middle areas (**B**), and 50% were in the apical area (**C**). Arrows indicate fractures.

The fracture load of the TRINIA group was 1555.9 ± 231.8 N, whereas that of the conventional fiber post group was 1082.1 ± 226.7 N (Table 2). The fracture load of the TRINIA group was 43.8% higher than that of the conventional fiber post group *(p* < 0.05). All specimens in the TRINIA group demonstrated cervical fracture. However, 37.5% of the specimens in the conventional fiber post group demonstrated cervical fracture and 50% of the specimens had apical fractures. Representative images of micro-CT are shown in each group (Figure 4). In the TRINIA group (A), all the specimens fractured in the cervical and middle areas and most of them were repairable even after fracture. In the conventional fiber post group, half of the specimens fractured in the cervical and middle areas of the resin core (B), and the other half did in the apical area (C).

## 4. Discussion

Fiber-reinforced materials have become popular in dental practice, particularly in restoring pulpless teeth with a post-core [8,9] and in the framework used for bridge restoration [10,11]. Fiber-reinforced resin composites that contain randomly distributed short glass fibers (0.2–0.3 mm) demonstrate higher flexural strength, elastic modulus, and fracture toughness compared with conventional resin composites [12]. Furthermore, fiber-reinforced resin composites containing long glass fibers (1–2 mm) have also been reported to have superior mechanical properties [13]. However, such fiber-reinforced resin composites have the disadvantage of limited glass fiber content, owing to the difficulty composites encounter due to high fiber content. To overcome this problem, we focused on a fiber-reinforced CAD/CAM resin disk containing 55 wt.% (45 vol.%) glass fibers. This is an innovative and original idea to produce a post-core from the fiber-reinforced CAD/CAM resin disk.

The results of the fracture test demonstrated that the fracture load of the TRINIA group was increased by approximately 43.8% relative to the conventional fiber post group. Regarding the fracture mode, the fractures in the TRINIA group initiated from the cervical regions, while the fractures of the conventional fiber post group propagated from the resin composite or the apical region (Figure 4). In particular, the clinical significance was that all the TRINIA specimens were fractured at the cervical region, suggesting that the fractured teeth were repairable, while 50% of the conventional fiber post group showed fracture at the root apex, and therefore was not recoverable. Therefore, the null hypothesis of this study was rejected.

In vitro fracture tests using extracted human or bovine teeth have been performed to evaluate the usefulness of the various restorative methods for pulpless teeth [14,15]. Because there are large individual differences in size, morphology, and calcification that makes the experimental values in human teeth less consistent, bovine teeth have been used in the present study with the expectation of small individual differences. Since bovine teeth frequently have enlarged root apexes that may affect their fracture resistance, teeth 30 months old with an apical size up to 1.3 mm were selected to obtain consistent results. The fracture load of TRINIA group had greater load bearing capacity compared with human central incisal restored by lithium silicate crown and short fiber-reinforced composite core [16] even under different experimental conditions. This suggests that the fiberglass woven into a mesh and layered structure of TRINIA is effective to reinforce pulpless teeth.

As for the adaptation of the post-core, the cement thickness of the CAD/CAM resin post-core has been greater than that of the metal post-core [17]. The cement thickness of TRINIA at the apical region possibly become greater than that of the fiber post because of the scanning difficulty. To improve adaptability of the CAD/CAM resin post-core, further investigation such as the use of another impression approach alternative to wax technique or the size adjustment of CAD model of the post-core should be conducted.

A ferrule is usually a recommended design for restoring a pulpless tooth with a post-core. However, in daily clinic applications, we often encounter extreme situations of patients having very limited remaining tooth structure. This study aimed to identify effective restoring methods, such as thin root dentin and no-ferrule at the cervical portion, for strengthening vulnerable pulpless teeth. Further studies of fatigue fracture tests under dynamic loading in various environments and remaining tooth structures are needed to evaluate the long-term durability in various oral environments.

## 5. Conclusions

In this study, the restored teeth with CAD/CAM fiber post-cores and zirconia crowns showed higher fracture resistance than those with conventional indirect fiber post-cores, and the restored teeth with CAD/CAM resin post-cores were found to be repairable even after fracture. These results suggest that the CAD/CAM resin post-core has the potential to be used clinically for preserving vulnerable pulpless teeth.

## Figures and Tables

**Table 1:** Compositions of the experimental materials.

Post-Core Materials		
TRINIA (SHOFU) Lot No. 037970914	glass fiber: 55 wt.%, epoxy matrix resin: 45 wt.%	
Beauti core flow paste (SHOFU) Lot No. 111611	Bis-GMA, TEGDMA, barium glass fillers	
Fiber post (SHOFU)	glass fiber, epoxy resin	
**Adhesive for TRINIA disk**		
HC primer (SHOFU) Lot No. 031601	UDMA, MMA, acetone, initiator	
**Dentin adhesive**		
ResiCem primer (SHOFU) Lot No. 061510 Lot No. 061511	Primer A	purified water, acetone, initiator
	Primer B	2-HEMA, 4-AET, acetone
**Resin cement**		
ResiCem paste (SHOFU) Lot No. 031862	Paste A	UDMA, TEGDMA, fluoroaluminosilicate, initiator
	Paste B	UDMA, TEGDMA, fluoroaluminosilicate, 4-AET,2-HEMA, initiator

Bis-GMA: bisphenol A-glycidyl methacrylate, TEGDMA: triethylene glycol dimethacrylate, UDMA: urethane dimethacrylate, MMA: methyl methacrylate, HEMA: hydroxyethyl methacrylate, 4-AET: 4-acryloxyethyl trimellitic acid.

**Table 2:** Fracture Loads and Patterns of Bovine Roots Restored with Fiber-Reinforced Post-Cores and Zirconia Crowns.

Fracture Patterns	TRINIA Group	Conventional Fiber Post Group
Cervical~middle	8	3
Middle~apical	0	1
Apical	0	4

## Data Availability

The data can be provided by the corresponding author on request.
